# Supplementary material for: Stochastic processes dominate the community assembly of ectomycorrhizal fungi associated with Betula platyphylla in Inner Mongolia, China
Source: PeerJ. 2025 May 19;13:e19364. doi: 10.7717/peerj.19364 (PMC12097238; doi:10.7717/peerj.19364)
Supplement: Supplemental Information 1 — Ectomycorrhizal fungal ecological processes. Pie chart of community construction process calculated by —RC_bary— [file peerj-13-19364-s001.pdf]

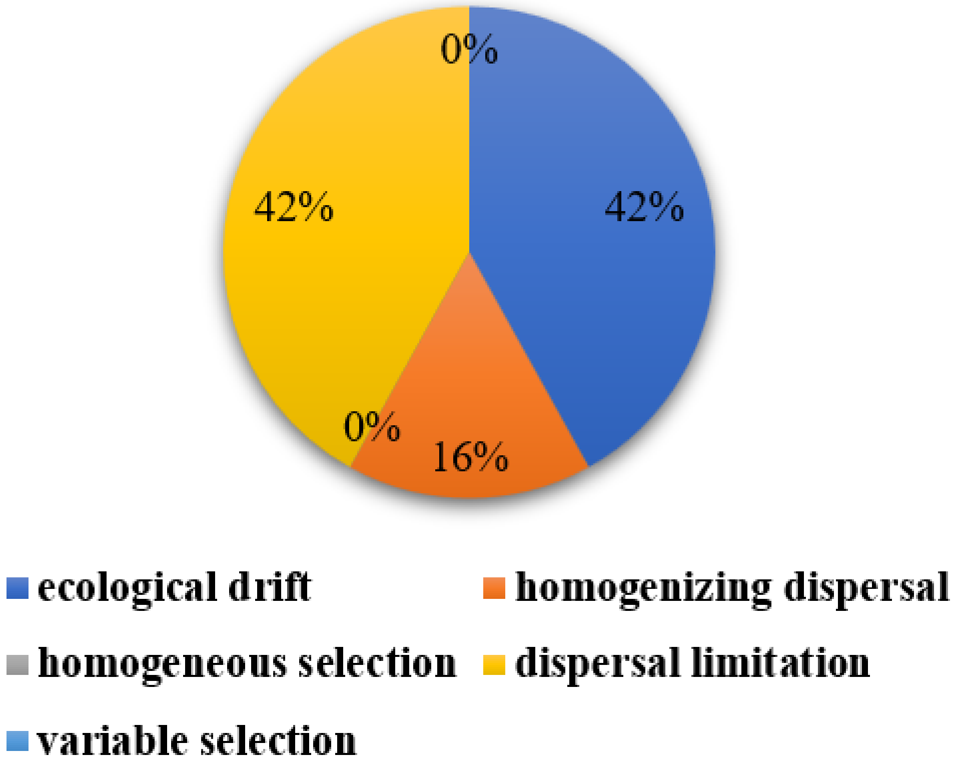

0%
42%
42%
0%
16%
ecological drift
homogenizing dispersal
homogeneous selection
dispersal limitation
variable selection
