# Supplementary material for: Stochastic processes dominate the community assembly of ectomycorrhizal fungi associated with Betula platyphylla in Inner Mongolia, China
Source: PeerJ. 2025 May 19;13:e19364. doi: 10.7717/peerj.19364 (PMC12097238; doi:10.7717/peerj.19364)
Supplement: Supplemental Information 5 [file peerj-13-19364-s005.docx]

**Supplementary Table 4 Correlations of spatial, soil and climatic variables with non-metric dimensional scale (Bray– Curtis distance) revealed by environmental fitting test.**

| Variable | NMDS1 | NMDS2 | *R*^2^ | *P* |
| --- | --- | --- | --- | --- |
| TOC | 0.39011 | -0.92077 | 0.9049 | 0.001 |
| altitude | -0.09083 | 0.99587 | 0.737 | 0.002 |
| AK | -0.80742 | -0.58998 | 0.7204 | 0.002 |
| AP | 0.27118 | -0.96253 | 0.7855 | 0.002 |
| MAT | 0.27131 | 0.96249 | 0.5214 | 0.007 |
| TN | 0.74621 | -0.66571 | 0.5321 | 0.014 |
| pH | 0.05235 | 0.99863 | 0.4947 | 0.023 |
| SWC | -0.13269 | -0.99116 | 0.4276 | 0.035 |

**Note: TN, soil total nitrogen; TOC total organic carbon; AK, available potassium; AP, available phosphorus; SWC, soil water content; MAT, mean annual temperature.**
